# Supplementary material for: Enhancement of Neuroblastoma NK-Cell-Mediated Lysis through NF-kB p65 Subunit-Induced Expression of FAS and PVR, the Loss of Which Is Associated with Poor Patient Outcome
Source: Cancers (Basel). 2021 Aug 29;13(17):4368. doi: 10.3390/cancers13174368 (PMC8430542; doi:10.3390/cancers13174368)
Supplement: Supplementary file 1 [file cancers-13-04368-s001.zip › cancers-1322675--SI.pdf]

Supplementary Materials: Enhancement of Neuroblastoma NK Cell-Mediated Lysis through NF-kB p65 Subunit-Induced Expression of FAS and PVR, the Loss of Which Is Associated with Poor Patient Outcome

Elisa Brandetti, Chiara Focaccetti, Annalisa Pezzolo, Marzia Ognibene, Valentina Folgiero, Nicola Cotugno, Monica Benvenuto, Paolo Palma, Vittorio Manzari, Paolo Rossi, Doriana Fruci, Roberto Bei and Loredana Cifaldi

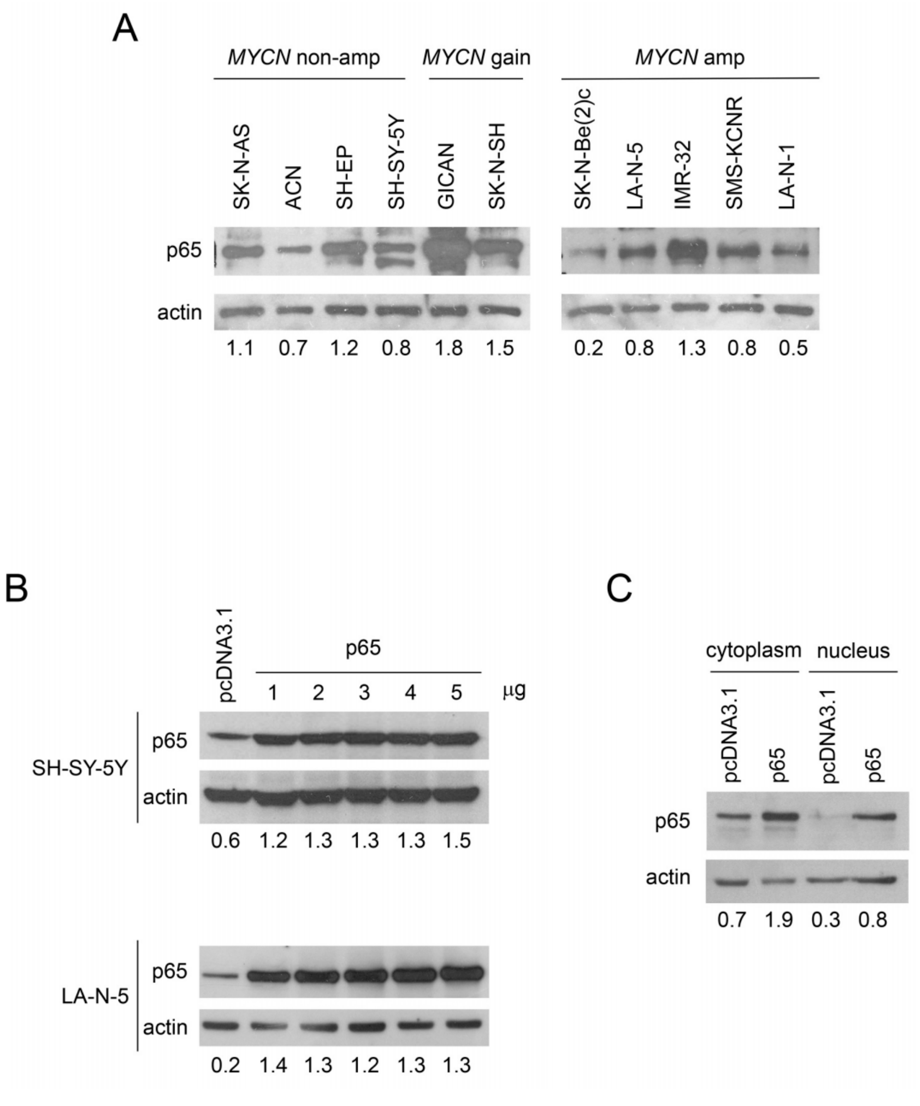

Figure S1. NB cell lines, nonamplified, gained and amplified for *MYCN* were analyzed for p65 subunit expression by western blotting. A representative of two independent experiments is reported. NB cells were transiently transfected with either an empty pcDNA3 vector or the indicated amount of vector expressing the p65 subunit for 48 h and analyzed by westernblotting. Expression of p65 LA-N-5 transfected with pcDNA3 or p65 was evaluated in both the cytoplasm and in nucleus by western blotting. A representative of three independent experiments was reported for each panel. Below each panel, the normalized densitometry, with respect to actin expression used as housekeeping, measured by ImageJ analysis is reported.

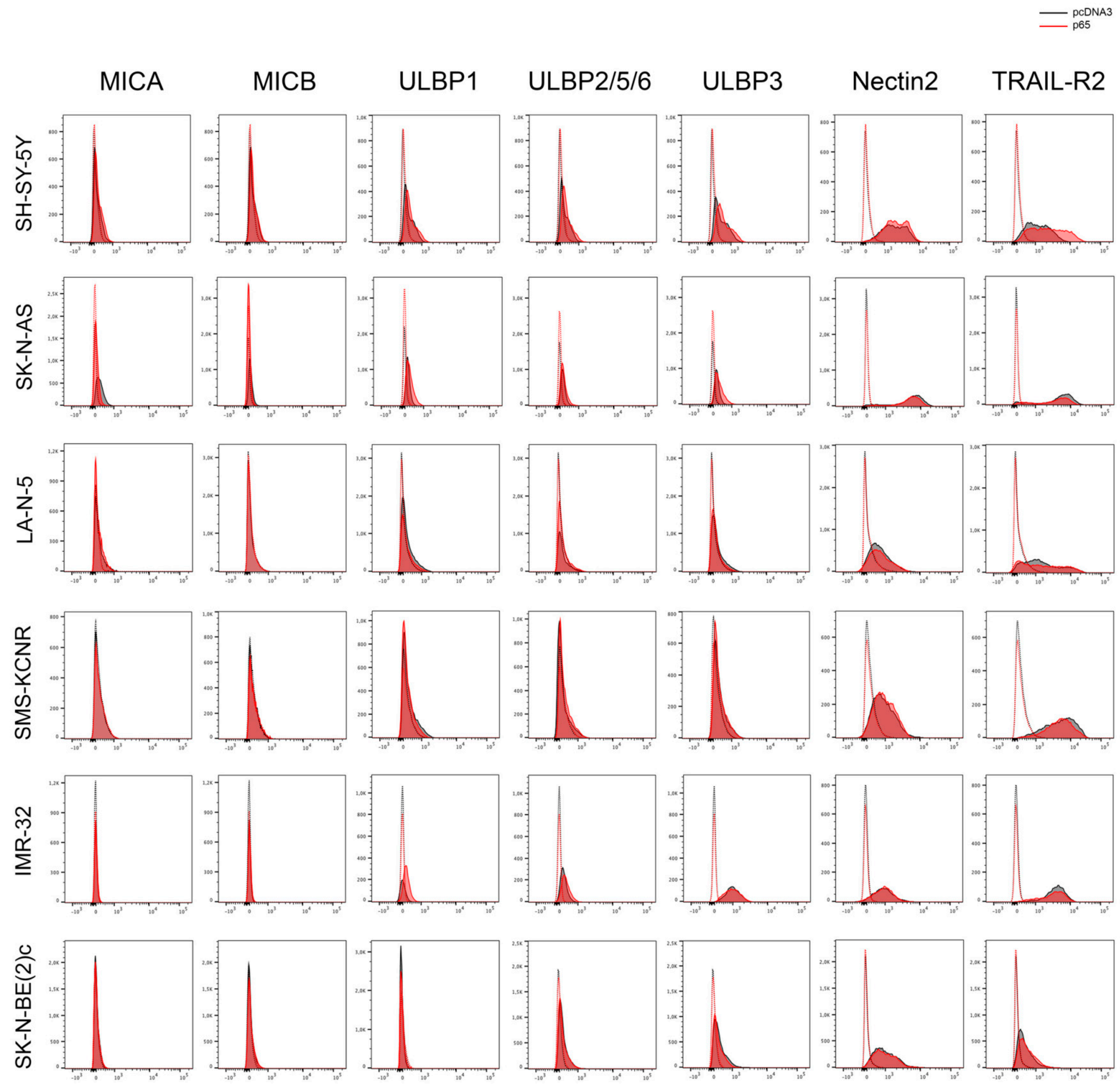

Figure S2. NB cell lines were transiently transfected for 48 h with either the empty vector pcDNA3 or the vector expressing the p65 subunit and evaluated for the surface expression of the indicated ligands for NK cell-activating receptors by flow cytometry analysis. Isotype-matched negative control antibodies are displayed as dashed gray and red lines for pcDNA3- and p65-transfectetd NB cell lines, respectively. A representative experiment out of the six performed is shown.

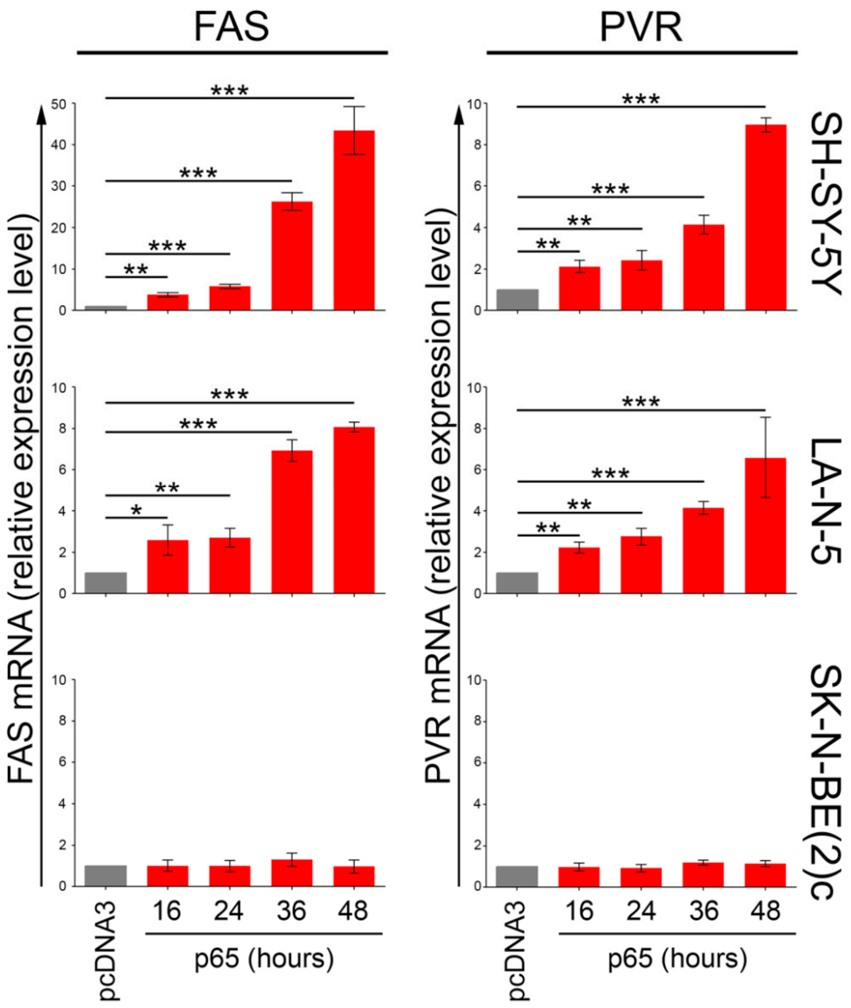

Figure S3. *FAS* and *PVR* mRNA evaluated by qPCR in NB cell lines transfected with the empty pcDNA3 vector or the vector expressing the p65 subunit at the indicated time points. Summary of four independent experiments is shown as mean ± SD; * $p < 0.05$; ** $p < 0.01$; *** $p < 0.001$.

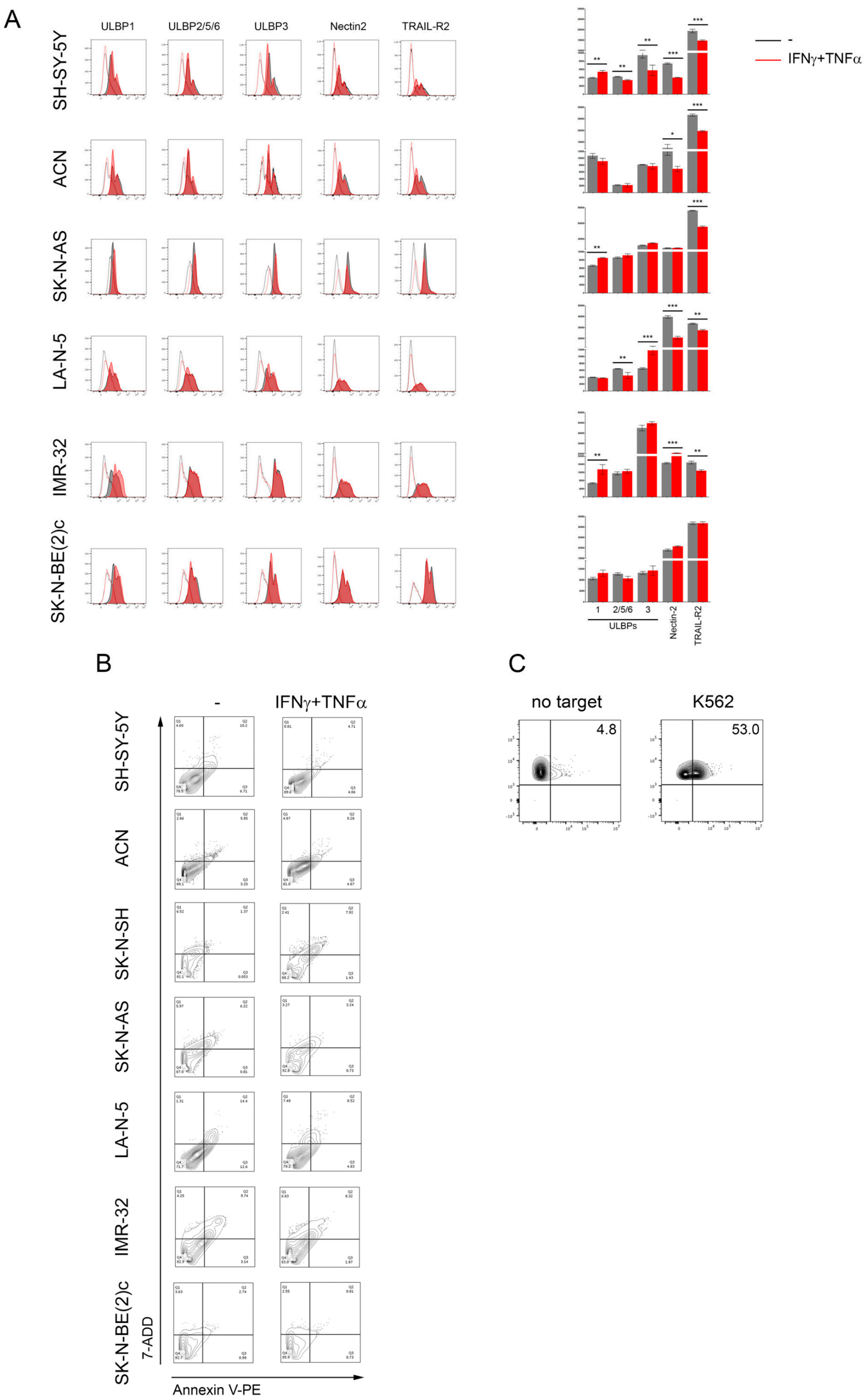
A
ULBP1
ULBP2/5/6
ULBP3
Nectin2
TRAIL-R2
SH-SY-5Y
ACN
SK-N-AS
LA-N-5
IMR-32
SK-N-BE(2)c
-
IFNγ+TNFα
1
2/5/6
3
ULBPs
Nectin-2
TRAIL-R2
B
-
IFNγ+TNFα
SH-SY-5Y
ACN
SK-N-SH
SK-N-AS
LA-N-5
IMR-32
SK-N-BE(2)c
7-ADD
Annexin V-PE
C
no target
K562
4.8
53.0

Figure S4. IFNγ- and TNFα-treated NB cell lines were evaluated for the surface expression of the indicated ligands by flow cytometry. Isotype-matched negative control antibodies are displayed as dashed gray and red lines for untreated and cytokine-treated NB cell lines, respectively. A representative experiment out of the 4 performed (left panel) and their summary, reported in histograms, (right panel) are shown; ** $p < 0.01$, *** $p < 0.001$. Untreated and cytokine-treated NB cells were evaluated for the apoptotic state by flow cytometry. A representative experiment, out of 4 performed, for each tested NB cell line indicated is reported. The percentage of cells in early phase (Annexin V^+ 7-ADD^-), and late phase (Annexin V^+ 7-ADD^+) of apoptosis, as well as dead cells (Annexin V^- 7-ADD^+) are reported for each plot. Representative NK cell-mediated degranulation in response to none or K562, as negative and positive control respectively, for the experiments shown in Figure 3D.

original western blotting for Fig S1A

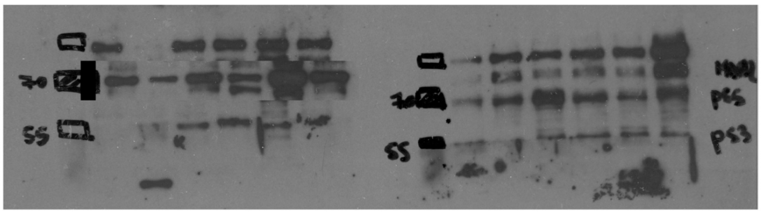

original western blotting for Fig S1B

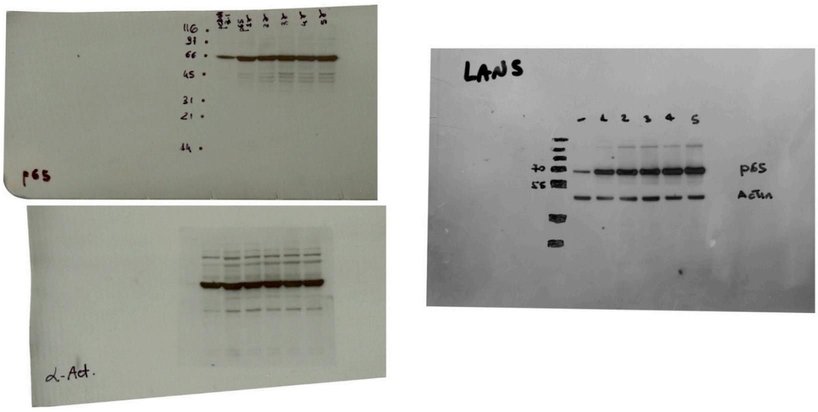

original western blotting for Fig S1C

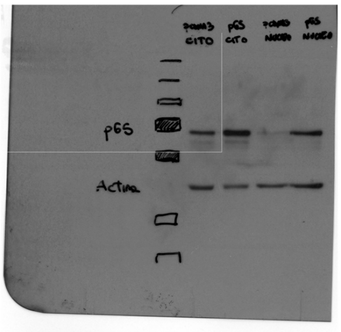

original western blotting for Fig 3A

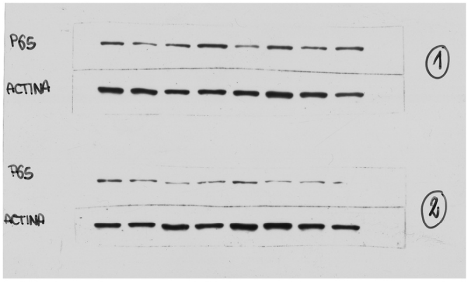

original western blotting for Fig 3E

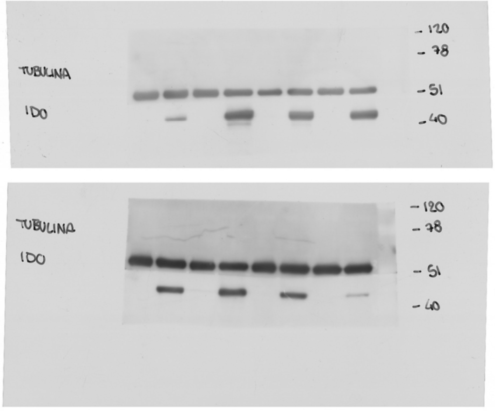

Figure S5. The original western blotting reported in Figure S1 and Figure 3 are shown as indicated.

Table S1. Status of *RELA* gene in NB patients listed in Table 1.

Case n°	Age at Onset	INSS Stage	INRG Stage	*RELA* Gene Status (11q13.1)	Relapse	Follow-Up	Disease State
1	2y 9m	3	L2	sc	yes	dead	
2	1y 10m	4	M	sc	yes	dead	
3	3y 11m	4	M	gain	yes	dead	
4	3y 9m	4	M	sc	yes	dead	
5	0y 10 m	3	L2	sc	yes	dead	
6	3y 1m	3	L2	sc	yes	dead	
7	3y 10m	3	L2	sc	yes	dead	
8	1y 2m	1	L1	sc	yes	dead	
9	3y 1m	1	L1	sc	yes	dead	
10	3y 4m	3	L2	sc	yes	alive	AD
11	5y 5m	3	L2	sc	no	alive	AD
12	16y 8m	2A	L1	sc	yes	alive	CR
13	4y 5m	2B	L2	sc	yes	alive	CR
14	4y 7m	4	M	sc	no	dead	
15	2y 5m	4	M	gain	yes	dead	
16	4y 4m	4	M	sc	yes	dead	
17	4y 5m	4	M	sc	yes	dead	
18	0y 7m	4	M	sc	yes	dead	
19	5y 2m	4	M	gain	yes	alive	AD
20	0y 1m	3	L2	sc	yes	dead	
21	6y 10m	2A	L1	sc	yes	dead	
22	1y 2m	1	L1	sc	yes	dead	
23	3y 9m	4	M	sc	yes	alive	CR
24	4y 4m	4	M	sc	no	alive	CR
25	1y 8m	4	M	sc	no	alive	CR
26	0y 4m	4s	Ms	sc	no	alive	CR
27	2y 10m	1	L1	gain	no	alive	CR
28	1y 2m	1	L1	sc	no	alive	CR
29	8y 1m	1	L1	sc	no	alive	CR
30	0y 4m	1	L1	sc	no	alive	CR
31	0y 8m	1	L1	sc	no	alive	CR
32	0y 11m	1	L1	sc	no	alive	CR

Table S2. Association between early and advanced stage of disease in NB patients with intact or loss of *FAS* and/or *PVR* genes.

Status of *FAS* and/or *PVR* Genes	NB Patients in Early Stages (83 in Stage 1 and Stage 2)	NB Patients in Advanced Stages (197 in Stage 3 and Stage 4)	*p*
intact (258)	77/258 (29.8%)	181/258 (70.1%)	ns
loss (22)	6/22 (27.3%)	16/22 (72.7%)	ns

ns, not significant *p* value; Fisher exact test.
